# Machine Learning Using Digitized Herbarium Specimens to Advance Phenological Research

**DOI:** 10.1093/biosci/biaa044

**Published:** 2020-05-13

**Authors:** Katelin D Pearson, Gil Nelson, Myla F J Aronson, Pierre Bonnet, Laura Brenskelle, Charles C Davis, Ellen G Denny, Elizabeth R Ellwood, Hervé Goëau, J Mason Heberling, Alexis Joly, Titouan Lorieul, Susan J Mazer, Emily K Meineke, Brian J Stucky, Patrick Sweeney, Alexander E White, Pamela S Soltis

**Affiliations:** California Polytechnic State University, San Luis Obispo, California; Florida Museum of Natural History, Gainesville, Florida; Department of Ecology, Evolution, and Natural Resources, Rutgers, the State University of New Jersey, New Brunswick, New Jersey; AMAP, the University of Montpellier and with The French Agricultural Research Centre for International Development, Centre National de la Recherche Scientifique, Institut National de la Recherche Agronomique, Institut de Recherche pour le Développement, Botanique et Modélisation de l’Architecture des Plantes et des végétations in Montpellier, France; Florida Museum of Natural History, the University of Florida, Gainesville, Florida; Harvard University Herbaria, Cambridge, Massachusetts; US National Phenology Network and with the University of Arizona, Tucson, Arizona; Natural History Museum of Los Angeles County, La Brea Tar Pits and Museum, Los Angeles, California; AMAP, the University of Montpellier and with The French Agricultural Research Centre for International Development, Centre National de la Recherche Scientifique, Institut National de la Recherche Agronomique, Institut de Recherche pour le Développement, Botanique et Modélisation de l’Architecture des Plantes et des végétations in Montpellier, France; Carnegie Museum of Natural History, Pittsburgh, Pennsylvania; Inria Sophia-Antipolis, Zenith team, Laboratoire d’Informatique, de Robotique et de Microélectronique de Montpellier (LIRMM), Montpellier, France; Inria Sophia-Antipolis, Zenith team, Laboratoire d’Informatique, de Robotique et de Microélectronique de Montpellier (LIRMM), Montpellier, France; Department of Ecology, Evolution, and Marine Biology, the University of California, Santa Barbara, Santa Barbara, California; Department of Entomology and Nematology, the University of California, Davis, Davis, California; Florida Museum of Natural History, the University of Florida, Gainesville, Florida; Yale Peabody Museum of Natural History, New Haven, Connecticut; Department of Botany and the Data Science Lab, the Smithsonian Institution, Washington, DC; Florida Museum of Natural History and with the University of Florida Biodiversity Institute, the University of Florida, Gainesville, Florida

**Keywords:** phenology, machine learning, biodiversity, climate change, deep learning

## Abstract

Machine learning (ML) has great potential to drive scientific discovery by harvesting data from images of herbarium specimens—preserved plant material curated in natural history collections—but ML techniques have only recently been applied to this rich resource. ML has particularly strong prospects for the study of plant phenological events such as growth and reproduction. As a major indicator of climate change, driver of ecological processes, and critical determinant of plant fitness, plant phenology is an important frontier for the application of ML techniques for science and society. In the present article, we describe a generalized, modular ML workflow for extracting phenological data from images of herbarium specimens, and we discuss the advantages, limitations, and potential future improvements of this workflow. Strategic research and investment in specimen-based ML methods, along with the aggregation of herbarium specimen data, may give rise to a better understanding of life on Earth.

Seasonal variation in the timing of life cycle (phenological) events (e.g., leaf-out, flowering, and fruiting) critically affects plant ecology and evolution through interactions between plants and climate (Cleland et al. 2007), herbivores (Meineke et al. 2014), mutualists (Diamond et al. 2014), and inter- and intraspecific competitors (Heberling et al. 2019). Changes in the timing of plant phenological events can alter species interactions, such as those between plants and their pollinators (Burkle et al. 2013) and even disrupt interactions at higher trophic levels, such as those between bears and salmon (Deacy et al. 2017). The significance and complexity of plant phenology on local and global scales lead to many compelling questions for science and society (Ellwood et al. 2019), especially as anthropogenic changes in habitat, biodiversity, and climate alter phenological events (Parmesan and Yohe 2003).

Wide-ranging and complex phenological responses to climate change have been discovered in many taxa across the globe (Willis et al. 2017a), but our understanding of the mechanisms driving these complicated phenomena and their subsequent effects are incomplete. Critical questions remain about the effects and interactions of climate, traits, geography, and phylogeny on the phenological sensitivities of plants and how these effects propagate throughout ecosystems (box 1). These factors could play key roles in understanding, predicting, and potentially ameliorating environmental changes that threaten biodiversity and humankind.

**Box 1. Example questions to advance the study of plant phenology using machine learning approaches.**

**Phenological research questions well suited for ML-based analyses**

The following list contains ecoevolutionary questions that require large sample sizes or, potentially, fine-scale data collection, rendering manual collection of phenological data from herbarium specimens infeasible.

- How is morphological variation related to phenology?
- Within or across species, do male and female flowers or individuals have different phenological responses to climate variables?
- Do closely related species differ in their phenological sensitivity to climate? If so, are such differences related to differences between taxa in habitat, climatic tolerance, geographic distribution, mean flowering time, or nonphenological traits?
- Do species with unisexual flowers differ in their phenological responsiveness from species with cosexual (i.e., perfect) flowers?
- How can certain phenophases be quantified in a way that is useful for predicting seasonal allergens, e.g., maturation of male catkins?
- How do global events affect regional phenology?
- What and where are phenological dark data (i.e., taxonomic, geographic, or other groups for which little phenological data are available)?
- How do climate and other factors affect how many flowers in an inflorescence become fruits (i.e., reproductive output)?
- How do climate and other factors affect the sizes and shapes of reproductive structures?
- How does the timing of phenological events and species-specific cues vary with latitude and across the globe? Do these interactions vary with climate change?
- How do nonnative species differ from native species with respect to phenological or distributional responses to climate change?
- How does nonangiosperm phenology (e.g., pollen release dates of gymnosperms) respond to climate and other factors?
- How do changes in plant phenology affect interactions between plants and other communities or trophic levels, including insects, pests, birds, and mammals?

**Questions about ML methodology**

- This list consists of questions relating to the methodology and limitations of machine learning for assessing phenology from herbarium specimens.
- Can ML algorithms reliably recognize plant reproductive structures?
- Can ML methods appropriately convey the level of confidence or uncertainty associated with any given classification or count?
- How many training images are necessary to train phenological data sets, and does this number exceed the number of specimens needed to conduct phenological studies?
- How does a computer “decide” how to classify specimens, and is the decision process generalizable?
- What are systematic errors from ML that could propagate and impact studies that use automatically annotated data?
- At which taxonomic scales and physical dimensions can ML contribute to phenological studies?
- How can the robustness of phenological annotations with ML approaches be ensured in data-deficient contexts (e.g., rare species or neglected floras)?

The variable and context-dependent nature of plant phenology renders its study particularly challenging because long-term data sets are necessary to detect the patterns, causes, and consequences of phenological change for a given taxon in a given location (Wolkovich et al. 2012). Historical phenological observations (Primack and Miller-Rushing 2012), agricultural records (e.g., crop harvest records; Ellwood et al. 2014), long-term field stations (Taylor et al. 2018), and, more recently, citizen science networks (Bison et al. 2018) have proven vital sources of phenological data. However, none of these resources provides the spatiotemporal coverage available from herbarium specimens. Herbarium specimens have been archived for hundreds of years from across the globe and therefore represent a rich source of phenological data (Zalamea et al. 2011), especially as specimen data (e.g., date, species, and locality) and specimen images become digitally available through large-scale digitization projects (Nelson and Ellis 2018). Specimen-based studies have greatly informed our understanding of phenological change beyond what was previously possible (Willis et al. 2017a).

To date, most phenological studies using herbarium specimens have relied on manual annotation (see table 1 for a glossary of italicized terms) of specimens to record phenological traits (Willis et al. 2017a). Herbarium staff might record the presence or absence of reproductive structures while transcribing specimen data, or, more commonly, researchers classify phenological traits from physical specimens or specimen images for specific projects. Such methods are time and labor intensive, and the accuracy and precision of the resulting phenological annotations depend on the botanical expertise and consistency of the scorer, as well as on how easily the relevant anatomical structures can be identified on dried, pressed plants. To study phenology on a global scale, many thousands—even millions—of specimens must be annotated, but manual annotation at this scale is not feasible. Machine learning (ML) approaches have the potential to overcome this challenge by automating phenological data acquisition. ML approaches have empowered advances in many areas of science and technology, with applications from self-driving cars (Wulff et al. 2018) to biomedical imaging (Ronneberger et al. 2015). Plant phenology, one of the best-known indicators of climate change, presents a fast-growing frontier for the deployment of these powerful methods.

**Table 1.** Glossary of machine learning (ML) terms discussed in this article.

Term	Definition
Annotation	Data added to a specimen record that is ancillary to the original collection data; annotations include, for example, phenological status, taxonomic identification, and georeferenced coordinates of a specimen; or the process of adding such ancillary data to a specimen record
Bounding box	(Often rectangular) area on a two-dimensional image that contains all points belonging to a given object; the sides of this area are defined by the extreme edges of the object
Convolutional neural network (CNN)	Type of neural network especially suited for image analysis that automatically learns relevant filters; other approaches use handcrafted filters and handcrafted feature extractors
Cyberinfrastructure	Computational environment and personnel resources which allow a combination of several tasks, including data acquisition, storage, management, mining, visualization, and analysis
Deep learning	Subset of machine learning techniques consisting of neural network models with a high number of successive layers; such models have been especially successful in tackling image, sound, and text tasks
Domain adaptation	Field of machine learning in which a model trained on a certain set of training data is co-opted for a different, but somewhat related, target data set
Filter	In image analysis, a specific type of local operation, i.e., a convolution, applied on the neighborhood of a pixel, designed to remove unwanted features from the image while preserving relevant information to perform a task, e.g., edge detectors for classification purposes
Graphics processing unit (GPU)	Hardware originally designed to accelerate computer graphics operations before being used for other kinds of computations; used heavily in deep learning computations
Instance segmentation	In image analysis, the task of finding the objects present in an image and their segmentation mask
Machine learning (ML)	Wide class of methods dedicated to the problem of training a computer to automatically learn to make predictions about novel data using annotated samples from a training data set
Metadata	Data used to describe or augment existing data
Object detection	In image analysis, the task of finding the objects present in an image and their bounding box
Plant phenology ontology (PPO)	The structured vocabulary for describing plant phenological observations that was developed to allow harmonization of data across disparate phenological data sets, including those from herbarium specimens (Stucky et al. 2018, Brenskelle et al. 2019)
Prediction	The output of a learning model, which is used to predict the most likely value or a mask for a new image
Segmentation mask	Defined path around an object containing exactly all the pixels of an image corresponding to that object, and only them
Training data or data set	Subset of the target data set to be annotated that is used to train ML algorithms according to the annotation schema; the training data set must already be annotated and should ideally contain a representative sample of the visual variation in the target data set
Transfer learning	Using knowledge gained from one domain of machine learning to improve another domain

ML algorithms build statistical models from input data (i.e., training data), and these models can then be applied to make predictions on novel data (LeCun et al. 2015). For image annotation tasks, for example, ML algorithms create statistical models (“learn”) from a training data set of images that have already been annotated. Then, the algorithms use these models to predict annotations for images that have not been annotated. ML has been successfully applied to many biological studies requiring classification of visual information, including the recognition and classification of animals in camera trap images (Norouzzadeh et al. 2018) and the automated species identification of herbarium specimens (e.g., Unger et al. 2016, Carranza-Rojas et al. 2017). Successful application of ML techniques to phenological annotation tasks has only recently been demonstrated for herbarium specimen images (Lorieul et al. 2019). In the present article, we describe a generalized ML workflow for phenological annotation of plant specimen images, and we discuss the advantages, limitations, and potential future improvements of this workflow. Furthermore, we explore how technological advances in ML will facilitate the collection of additional phenological and other trait data from images, enhancing ecoevolutionary research and biodiversity education.

## Machine learning with digitized herbarium specimens

**Figure 1.**
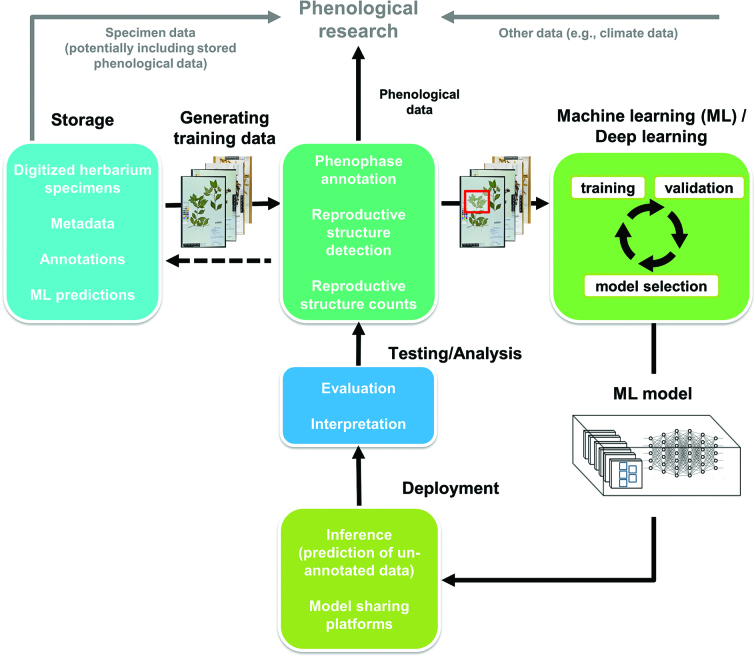
Key components of a generalized, modular machine learning (ML) workflow applied to the annotation of herbarium specimen images for phenological traits. Specimen images are retrieved from storage, and a representative subset of the focal images are used to generate a set of training data. The training data, which have been manually annotated according to the desired phenological scoring protocol (e.g., flowers present or absent), are used as input data for ML. The resulting statistical model is then deployed to predict phenological annotations for previously unannotated specimens. The accuracy and precision of the ML model(s) can be tested using a subset of manually annotated data to compare predicted annotations to those recorded by expert observers. Newly annotated specimens, combined with specimen label data, georeferenced localities, and other data sets (e.g., historical climate data), can then be used in an array of phenological research.

Figure 1 presents a workflow of five components for phenological annotation of herbarium specimen images (storage, generating training data, machine learning, deployment, and testing and analysis; figure 1), further described below. A detailed discussion of ML methodology can be found in Lorieul and colleagues (2019). In the present article, we use the term annotation to describe the general process of adding ancillary data to a specimen record. Although we focus on phenological annotation—recording of the phenological status of a specimen—many other types of annotation exist, such as georeferencing, taxonomic identification, and scoring of nonphenological traits.

Core specimen data, such as collection date, scientific name, collector name, and textual locality information, are often captured and mobilized as part of large-scale digitization initiatives (e.g., more than 120 million specimen records contain this information in iDigBio; www.idigbio.org). Therefore, we do not describe the use of ML for harvesting these data. We focus in the present article on the application of ML to automatically identify and record the phenological status of specimen images, ideally those images for which these core specimen data are already available.

### Storage

Input and output data for ML approaches consist of digital images of specimens, associated core specimen data (e.g., specimen identifier, collection date, etc.), and phenological annotations. High-resolution images (e.g., up to tens of millions of pixels per specimen) can represent a very large volume of data (4–10 megabytes per image), and their storage therefore requires software and hardware infrastructure designed for large amounts of data.

### Generating training data

The images used to “train” ML algorithms must first be manually annotated with phenological information according to the desired protocol. For example, if a researcher is interested only in whether a specimen has flowers, each specimen image would be annotated as flowers “present” or “absent.” A model based on these training data will classify specimens as either of these two classes. If the researcher wishes to detect individual reproductive structures on a specimen, for example, in order to count them or estimate their relative proportions, these could be delineated by bounding boxes, which indicate the general location of structures, or segmentation masks, which designate all pixels belonging to a reproductive structure. To produce these more complex annotations, specific annotation tools are usually required (e.g., CocoAnnotator, https://annotator.justinbrooks.ca; LabelMe, http://labelme.csail.mit.edu/Release3.0; ImageTagger, https://github.com/bit-bots/imagetagger). Regardless of the type of phenological annotation being created, it is important to recognize that ML is a form of statistical inference that finds patterns in the training data, and predictions will therefore reflect any biases in those data. Therefore, the training data set should include a representative sample of taxon (or taxa) and phenological attributes or categories that should appear in the data set on which the trained model will be applied. A lack of visual diversity in the manual annotations will result in the same lack of diversity in the predictions. Similarly, noisy, imprecise, or incomplete annotations may result in noisy, imprecise, or incomplete predictions. A subset of the training data should also be reserved for model validation (see the “Testing and analysis” section).

The number of specimens necessary to train a model depends on the complexity of the classification task, the number and morphological diversity of taxa included in the data set, and whether the model will be trained iteratively. Initial models with a small set of training data (at least 500 specimens) can be used to annotate a much larger set of data, and the ML-generated annotations can be revised and enriched through additional manual annotation, and then used to train the model further (see Affouard et al. 2017). As the annotated training data set grows, models will improve and become more useful for identifying edge cases (e.g., specimens on which phenological attributes are partially obscured, taxa that are represented by only a few specimens) that might be missing in a small training data set, as well as phenological phases that are morphologically highly variable or represented by very small reproductive structures.

### Machine learning and deep learning

For image data, the most effective machine learning techniques are deep learning neural networks, or more precisely, convolutional neural networks (CNN), or extensions such as CNNs for object detection (e.g., R-CNN) or instance segmentation (e.g., Mask-R-CNN). Like any neural network, a CNN is a composition of functions that receive an image as input and provide predictions as output (e.g., a phenological score of the entire specimen or a set of bounding boxes around reproductive structures). The term convolutional refers to the fact that for each input pixel, the functions return the result of a local computation (a filter) based on the pixel and its neighboring pixels. The number of neighboring pixels (i.e., filter size) is set as a parameter of the model. Each filter can be interpreted as a detector of a specific, local visual pattern.

CNNs consist of multiple layers of processing. The first layers of a CNN detect low-level patterns (color changes, angles, textures, etc.), whereas the last layers recognize complex visual patterns (such as reproductive structures on a specimen) by combining the patterns found in previous layers. The main objective of the training phase of a CNN model is to estimate the parameters of these filters on the basis of the training data. This is done by iteratively minimizing a loss function measuring the error between the predictions and the expected manual annotations.

As the number of parameters to be estimated can be in the millions, this training phase consumes significant computational resources, because the training process requires efficient storage to handle frequent access to the training set and efficient graphics processing units (GPUs) to reduce the training time. The type of model may differ depending on the task, but the duration of the training phase is strongly related to the GPU number and characteristics. The greater the availability of computational resources, the more training a model can undergo, and the better the final model architecture and parameter selection can be.

Available online tools can enable inexperienced users to train ML models, but these tools are limited to simple image classification tasks and low data volumes. Therefore, at present, developing accurate models is often achieved by involving a data scientist for several days, weeks, or months depending on the difficulty of the task. The software frameworks most commonly used to develop CNNs are Pytorch (https://pytorch.org), TensorFlow (www.tensorflow.org), CAFFE (https://caffe.berkeleyvision.org), and MXNET (https://mxnet.incubator.apache.org). The framework that is best for a given task will depend on a variety of factors, including the availability of preexisting models or code for the targeted task, the hardware used, or simply the data scientist's skills with respect to a particular framework.

### Deployment

Once an ML model is trained, it can be deployed to predict phenological annotations for previously unannotated specimens. This process could be implemented in several ways, including via a web service available to other applications that returns the model output for any submitted image, a standalone program, or an end-user graphical user interface (GUI) web application. Inference is usually a less computationally intensive task than training and therefore requires fewer resources. Models can be publicly shared through software repositories hosted on sites such as GitHub (https://github.com), GitLab (https://gitlab.com), or dedicated platforms such as Model Zoo (https://modelzoo.co). Such sharing encourages cross-model improvement based on transfer learning (Bengio 2012) or domain adaptation techniques (Long et al. 2016).

### Testing and analysis

Once deployed, a model can be evaluated through qualitative judgments or quantitative measurements. Qualitative judgments are typically performed through a GUI, allowing the researcher to visualize and assess the automated predictions of the model on this test data set. For more robust measures of model accuracy, quantitative measurements of the quality of the predictions can be readily calculated and visualized (see Lorieul et al. 2019). This step cannot be performed on the same data used for training the model; a model can perfectly fit the training data and not fit new data of the same type. Therefore, some of the manually annotated data (e.g., 20%–30%) must be reserved and not used for training purposes (Carranza-Rojas et al. 2017). As with developing appropriate training data, a deep understanding of the data set and the desired end product is necessary. The goals are to determine whether the model meets the needs of the researcher and to identify the necessary progress points (e.g., new annotations on a particular taxonomic group or phenological stage).

## Advantages of machine learning for phenology

The main advantage of using ML for phenological annotation is ML’s ability to score very large volumes of data in a short amount of time. Once an algorithm has been trained, it can score tens to thousands of specimens per minute, depending on the task, with a single standard GPU. For example, during summer 2019, more than 490,000 field plant images were analyzed for species identification on the public Pl@ntNet platform in a single day (Affouard et al. 2017). Model processing can be further accelerated by using many GPUs in parallel. Another key advantage is that the accuracy of ML annotations can be very high. The deep learning method evaluated in Lorieul and colleagues (2019) correctly detected fertile specimens with an accuracy of 96.3%. Such a success rate is quite high, especially considering that one coauthor of the present article obtained an accuracy of only 87.8% at the same task. Accuracy was slightly decreased for finer-scale phenological annotations (84.3% for the detection of flowers and 80.5% for the detection of fruits) or for determining specific phenophases of specimens, but again, the model's accuracy was slightly better than the accuracy of a human expert.

With the large volumes of phenological data produced from ML methods, it will be possible to create phenoclimatic and other phenological models at unprecedented scales of time, space, and phylogenetic diversity. Leveraging a large number of specimens for phenological models has already helped identify influential climate variables that may have been overlooked in previous studies (Park and Mazer 2018). Large sample sizes and potentially finer scales of phenological annotation could further elucidate differences in phenological trends among taxa and between regions. Phenological annotation via ML could also be used to address current spatial biases in phenological studies, for example, through applications using herbarium specimens from tropical and subtropical climates, where phenological trends are less well documented (Willis et al. 2017a).

## Limitations of machine learning applications

**Figure 2.**
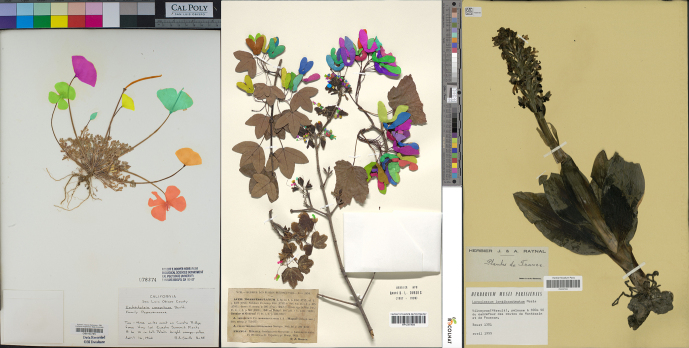
Examples of herbarium specimens displaying visual heterogeneity (e.g., in morphology, labels, and color standards) and challenges related to the morphology and position of reproductive structures. The specimen on the left shows large, isolated reproductive structures that would likely be annotated successfully by ML algorithms. The specimen in the center with small flowers and numerous overlapping fruits would be much more difficult for ML algorithms to parse. The specimen on the right would be very difficult for ML algorithms to delineate or count because of the unclear distinction between flowers and buds. Examples of segmentation masks (see the glossary in box 1) created to delineate reproductive structures are shown by the brightly colored areas in the left and center specimen images.

The workflow described above is not without limitations; there are technological, social, and logistical challenges that must be overcome for efficient application of ML in phenological research. Herbarium specimens may themselves limit their utility for phenological scoring, because they differ in quality and quantity (figure 2; for a review, see Willis et al. 2017a). The morphological structures necessary to measure a given phenological trait may be damaged or lost, stored in opaque fragment packets, or obscured by other plant material on the sheet. Some plant taxa have phenological stages that are impossible to determine without dissecting the physical specimen. For example, the fruits of sedges are often indistinguishable from female flowers, and fig flowers are tightly enclosed within vegetative receptacles. Furthermore, herbarium specimens often consist of only a portion of a plant, and therefore the reproductive status of the entire plant may not be readily discernible except via the label or field notes, when they exist. In each of these cases, ML algorithms, no matter how accurate at recognizing reproductive structures on herbarium specimen images, cannot fully determine the phenological status of a plant from a specimen.

Beyond these general issues, there are additional considerations for applying ML to herbarium specimen images. Specimens contain a significant amount of nonplant material such as herbarium stamps or logos, labels, mounting tape, color standard plates, rulers, or evidence of past pest-control measures that may bias certain ML classification tasks (figure 2). Because ML techniques use all visual data available, the algorithms could reflect differences in preparation techniques rather than in the presence or numbers of particular plant structures. Finally, when preparing training data sets, users of herbarium data must be aware of duplicate specimens—the same species collected at the same time in the same place—and of possible misidentified specimens.

## Future solutions for developing optimal machine learning workflows

Perhaps the greatest limitation of the ML workflow is the requirement for adequate training data sets. Training data sets need not be large, but a visually diverse set of specimen images of focal taxa must be available. Although several million herbarium specimens have been digitized and mobilized online (currently nearly 30 million images in iDigBio), most of the 375 million specimens in herbarium collections worldwide are not digitized. Continued digitization of herbarium specimens is needed, particularly of phylogenetically and morphologically diverse collections that can reflect visual variation among specimens and therefore enable the creation of more versatile training data sets.

As more specimen images become available, they must also be accurately annotated according to the desired phenological classification protocol. Citizen science and crowdsourcing can accelerate the image annotation process, as has been demonstrated by platforms such as Zooniverse (www.zooniverse.org), From the Page (https://fromthepage.com), Pl@ntNet (https://plantnet.org/en), and CrowdCurio (Willis et al. 2017b). Engaging students in this process as a learning experience could be a dual-benefit solution (see the “Extending machine learning” section). Mass annotation by volunteers with heterogeneous skills presents its own challenges, including the need to attract and retain volunteer interest and ensure data quality, but it offers the additional benefits of community engagement and education.

Regardless of how training data sets are created, both input and output data of ML analyses must be accessible, reproducible, and reusable. ML metadata, such as the type of model used to create the prediction, the contact information of the model creator, uncertainty regarding phenological predictions, and reference to the training data set, must be carefully documented so that downstream users can reproduce and assess the utility of the scorings for their specific research purposes. Furthermore, the potentially complex outputs of ML approaches (e.g., coordinates of reproductive structures on an image) must be stored in a way that is interoperable with alternative specimen classification methods (e.g., manual annotation) and intelligible to nonexperts of ML, while at the same time being reproducible by those with ML expertise. The development of standard vocabularies, metadata protocols, and data structures within the scaffold of existing biodiversity data standards (i.e., Darwin Core; see Yost et al. 2018) would greatly advance this aim. When designing ML-based phenological studies, researchers should consider how to integrate output data and metadata with existing databases or data sets, ideally using terms and relationships from the Plant Phenology Ontology (Stucky et al. 2018, Brenskelle et al. 2019). A relatively small investment early in the research process can ensure that ML-generated, “extended” data (sensu Lendemer et al. 2019) can be productively leveraged by other researchers and owners of the original data (i.e., natural history collections), thereby increase the value of the specimens. This process would benefit from further discussion across biological and computer science disciplines, for example, within the framework of the Research Data Alliance (www.rd-alliance.org).

Another significant advance for ML-based phenological annotation would be a better understanding of the limitations of ML methods for phenological annotation tasks. Current specimen-based studies using ML have yet to diversify beyond simple classification tasks (e.g., whole images classified as “flower present” or “flower absent”) to more complex tasks, such as object detection and localization (see Girshick et al. 2014) or the use of models that focus on a subset of visual data, rather than the entire image (see Mnih et al. 2014). To move forward with the design and implementation of ML techniques for phenological studies, questions concerning methodology and appropriateness (e.g., table 1) must be addressed with future research.

However, evaluating models with high complexity may not be easy to achieve given presently available, highly localized cyberinfrastructure. ML approaches, especially those using thousands of high-resolution images, require computers with fast GPUs, but high-power computing clusters are generally heavier in central processing units (CPUs) than GPUs. Therefore, having advanced computing resources does not necessarily guarantee the ability to conduct image-based ML experiments. Although publicly accessible GPU resources (e.g., XSEDE; Towns et al. 2014) and sharing of ML methods (e.g., through Model Zoo) are growing, large-scale and collaborative platforms for using and sharing ML techniques for biological applications are lacking. Greater investment in interoperable cyberinfrastructure resources that are available to a broader, collaborative community is needed.

Increased communication, collaboration, and sharing of cyberinfrastructure resources across institutional and disciplinary boundaries (e.g., between computer scientists and biodiversity data managers) are also critical to advancing this field. Fully exploring the potential of ML approaches may require collaboration beyond academia. Several private companies already provide financial or IT resources to advance ML techniques in the fields of biodiversity, agriculture, and the environment, including Microsoft, with the AI for Earth initiative, and Google, which supports workshops on the fine-grained visual categorization of various biological entities. Collaboration with technology companies such as FaceBook, Dell, or Nvidia, which have significant expertise and resources in this field, may be influential in the successful development and deployment of ML applications for large herbarium collections.

Many of the challenges in the phenological workflow outlined above are generalizable, and ML innovations from other disciplines hold promise for applications in herbarium science. Broadly, museum-based applications may benefit from innovations in ML model architectures in which tasks are general (e.g., segmenting images into constituent elements), transfer learning from other domains in which plant images may be used (e.g., agricultural applications), and ML-based applications in associated data-rich domains in which images are associated with rich tabular data (e.g., medical images, which may be linked to multidimensional patient histories) (Esteva et al. 2017). Advances in medical imaging in which specific anatomical structures need to be identified, measured, and evaluated for patient diagnosis have already provided the ML community with model architectures (Ronneberger et al. 2015) that are now applied broadly to segmentation tasks in domains as seemingly distant as automated driving (Wulff et al. 2018). Other disciplines that may yield useful innovations include astronomy, single-cell phenotyping, finance, e-commerce, manufacturing, and defense. Potentially useful botanical applications shared with these domains include anomaly detection, segmentation, high-dimensional image-based clustering, and digital transcription. Recent advances in digital agriculture (e.g., Ghosal et al. 2018) present a number of promising avenues for extension into phenological research, most importantly because visual models in agriculture are often trained to interpret images of plants and identify important plant structures and attributes (Ferentinos 2018).

Perhaps the most important aspect to improving ML applications for herbarium specimens is open discussion and collaboration between biological and computer science communities. This includes continued development of the community of biologists who are interested in applying these models to ecological and evolutionary questions, as well as finding appropriate avenues through which biologists can communicate pressing goals in biodiversity science to ML experts. Ultimately, these communities must also communicate effectively with end users of automatically scored specimen records, such as ecologists and collection managers, to promote understanding of ML-derived outputs and their limitations.

## Diversifying phenological research using machine learning

Most herbarium-based phenological research to date has focused on understanding the timing of key flowering events, especially first flowering or peak flowering, using fairly simple phenological annotation protocols (Willis et al. 2017a). However, many specimens contain multiple reproductive structures representing a gradient of phenological stages (e.g., flower buds and open flowers present). ML methods could be leveraged to quantify numbers of reproductive structures on specimens, which provides a more detailed understanding of phenological traits such as flowering duration, the rate of progression between phenophases, and phenophase-specific responses to climate change (Love et al. 2019).

The phenology of less well-studied taxonomic and regional groups such as bryophytes, ferns, gymnosperms, and taxa in tropical climates could also be accelerated by ML approaches. Furthermore, herbarium specimens can be used to assess nonreproductive phenological processes such as leaf-out (Everill et al. 2014). In some taxa, it may even be possible to track phenological patterns of primary growth such as stem elongation. ML-based methods could provide a reliable approach to annotating the vegetative phenology of specimens at a large scale, similar to those for reproductive phenology (table 1).

## Extending machine learning analysis of herbarium specimens beyond phenology

The current era of rapid advances in ML coincides with a similarly transformative era for biological collections, especially in global change research. Motivated by widespread museum digitization initiatives, herbarium specimens are increasingly being used in a variety of new ways in research (Meineke et al. 2018a, Hedrick et al. 2020) and in the effective engagement of the public through citizen science and education (e.g., Lacey et al. 2017). In the present article, we focused on phenology because phenological inference is an emerging tool with links to adaptation, population success, ecophysiology, carbon and nutrient dynamics, human health and sociocultural applications, and resource management. Many of these efforts could be expanded or transformed by the application of ML tools built to automate tasks because the approaches described above can be applied to a wide array of research questions. In particular, ML shows promise in the study of nonphenological traits, the study of plant interactions with nonplant taxa, and the engagement of the broader community to promote biodiversity literacy.

### Nonphenological traits

Herbarium specimens are rich with phenotypic data, and specimen images provide the potential for automated trait measurement. Specimens have long provided morphological characters (e.g., leaf shape, reproductive structures) used to identify plant taxa; however, these measurements were traditionally limited to species-level trait means or ranges for identification purposes only. Influenced by developments in functional trait-based ecology (e.g., Reich 2014) and advances in global change biology, specimen phenotypes are being used for studies of evolutionary and ecological change at large taxonomic, temporal, and geographic scales. For example, phenotypic data from specimens have been used to quantify rapid leaf trait evolution in invasive species following introduction (Buswell et al. 2011) and to document changes in plant size as a result of human harvesting (Law and Salick 2005) or climate change (Leger 2013). To scale up these studies, ML models could be developed and deployed to efficiently measure functional traits for thousands of herbarium specimens (high-throughput phenotyping; Gehan and Kellogg 2017). These traits could include leaf morphometric traits (size, shape), plant size (height, area), and inflorescence and floral traits (size, number).

### Species interactions

Data on species interactions are currently sparse, but hypotheses in this area of global change research are central to ecology and evolutionary biology. Herbarium specimens and other collections provide unique opportunities to quantify interactions across time and over large geographic ranges (Lees et al. 2011, Meineke et al. 2018b). ML-based approaches have the potential to broaden and deepen the scope of available data and therefore facilitate new discoveries with applications in conservation, ecology, and other fields. For example, ML could automate the recognition of leaf mines, damage from herbivores, or plant diseases (Ferentinos 2018, Ingram et al. 2017).

### Biodiversity literacy

ML methods and ML-generated data can provide educational opportunities beyond what is possible with specimens alone. ML has yet to be widely applied in biodiversity education, but on the basis of work using specimen-based data in undergraduate courses, such data can provide authentic introductions to scientific skill building, biodiversity and data literacy, and workforce training (Lacey et al. 2017 and the references within it). Educators can also integrate specimen annotation activities into coursework using online citizen science platforms such as Zooniverse. In addition, students can learn data management skills, investigate research questions of their own design, and gain experience with data analysis and visualization when working with the wealth of data generated from ML. Furthermore, it is possible to involve students in more technical aspects of the ML workflow, such as model creation and validation. The application of ML to digital specimen data provides an engaging, well structured, freely available introduction to data science.

## Conclusions

Machine learning offers an efficient approach to collecting large amounts of phenological data from herbarium specimens. When combined with, for example, spatiotemporal data extracted from specimen labels during digitization, these data enable discovery of phenological patterns on unprecedented scales. ML models can annotate thousands to millions of images in relatively short time spans, potentially with greater reproducibility and in finer detail than is feasible with human labor alone. Furthermore, the adaptability of ML models can empower specimen-based research beyond phenological traits, facilitating myriad avenues of biological research.

Despite these clear advantages, applying ML to specimen images has limitations and challenges, many of which could be overcome through research and development in several key areas. First, training data sets must be developed through continued digitization of herbarium specimens and annotation of specimen images. Second, research questions such as those in table 1 must be addressed to ensure ML-based methods are being effectively employed. Third, greater attention must be paid to downstream use of automatically generated data. Primary concerns include mapping ML-produced data to existing standards (e.g., Darwin Core, the Plant Phenology Ontology) and linking these data to existing specimen data (Lendemer et al. 2019). Finally, to fully realize the potential of ML approaches to phenology and biodiversity science, there is great need for collaborative cyberinfrastructure to manage large quantities of visual data, including the development of an interdisciplinary community aimed at synergistically advancing ML-based methods for science and society.

## References

[bib1] Affouard A, Goëau H, Bonnet P, Lombardo JC, Joly A 2017 Pl@ntnet app in the era of deep learning. ICLR 2017 Workshop Track: 5th International Conference on Learning Representations, Toulon, France.

[bib2] Bengio Y. 2012 Deep learning of representations for unsupervised and transfer learning. In Proceedings of ICML workshop on unsupervised and transfer learning. 17–36.

[bib3] Bison M, Yoccoz NG, Carlson BZ, Delestrade A 2018 Comparison of budburst phenology trends and precision among participants in a citizen science program. International Journal of Biometeorology 63: 61–72. 30382351 10.1007/s00484-018-1636-x

[bib4] Brenskelle L, Stucky BJ, Deck J, Walls R, Guralnick RP 2019 Integrating herbarium specimen observations into global phenology data systems. Applications in Plant Sciences 279: e01231. 10.1002/aps3.1231 PMC6426164 30937223

[bib5] Burkle LA, Marlin JC, Knight TM 2013 Plant-pollinator interactions over 120 years: Loss of species, co-occurrence, and function. Science 339: 1611–1615. 23449999 10.1126/science.1232728

[bib6] Buswell JM, Moles AT, Hartley S 2011 Is rapid evolution common in introduced plant species? Journal of Ecology 99: 214–224.

[bib7] Carranza-Rojas J, Goeau H, Bonnet P, Mata-Montero E, Joly A 2017 Going deeper in the automated identification of herbarium specimens. BMC Evolutionary Biology 17: 181. 28797242 10.1186/s12862-017-1014-z PMC5553807

[bib8] Cleland EE, Chuine I, Menzel A, Mooney HA, Schwartz MD 2007 Shifting plant phenology in response to global change. Trends in Ecology and Evolution 22: 357–365. 17478009 10.1016/j.tree.2007.04.003

[bib9] Deacy WW, Armstrong JB, Leacock WB, Robbins CT, Gustine DD, Ward EJ 2017 Phenological synchronization disrupts trophic interactions between Kodiak brown bears and salmon. Proceedings of the National Academy of Sciences 114: 10432–10437. 10.1073/pnas.1705248114 PMC5625906 28827339

[bib10] Diamond SE, Cayton H, Wepprich T, Jenkins CN, Dunn RR, Haddad NM, Ries L 2014 Unexpected phenological responses of butterflies to the interaction of urbanization and geographic temperature. Ecology 95: 2613–2621.

[bib11] Ellwood ER, Playfair SR, Polgar CA, Primack RB 2014 Cranberry flowering times and climate change in southern Massachusetts. International Journal of Biometeorology 58: 1693–1697. 24018848 10.1007/s00484-013-0719-y

[bib12] Ellwood ER, Pearson KD, Nelson G 2019 Emerging frontiers in phenological research. Applications in Plant Sciences 7: e1234.

[bib13] Esteva A, Kuprel B, Novoa RA, Ko J, Swetter SM, Blau HM, Thrun S 2017 Dermatologist-level classification of skin cancer with deep neural networks. Nature 542: 115. 28117445 10.1038/nature21056 PMC8382232

[bib14] Everill PH, Primack RB, Ellwood ER, Melaas EK 2014 Determining past leaf-out times of New England's deciduous forests from herbarium specimens. American Journal of Botany 101: 1293–1300. 25156979 10.3732/ajb.1400045

[bib15] Ferentinos KP. 2018 Deep learning models for plant disease detection and diagnosis. Computers and Electronics in Agriculture. 145: 311–318.

[bib16] Gehan MA, Kellogg EA. 2017 High-throughput phenotyping. American Journal of Botany 104: 505–508. 28400413 10.3732/ajb.1700044

[bib17] Ghosal S, Blystone D, Singh AK, Ganapathysubramanian B, Singh A, Sarkar S 2018 An explainable deep machine vision framework for plant stress phenotyping. Proceedings of the National Academy of Sciences 115: 4613–4618. 10.1073/pnas.1716999115 PMC5939070 29666265

[bib18] Girshick R, Donahue J, Darrell T, Malik J 2014 Rich feature hierarchies for accurate object detection and semantic segmentation. IEEE Conference on Computer Vision and Pattern Recognition. IEEE doi:10.1109/CVPR.2014.81

[bib19] Heberling JM, McDonough MacKenzie C, Fridley JD, Kalisz S, Primack RB 2019 Phenological mismatch with trees reduces wildflower carbon budgets. Ecology Letters 203: 616–623. 10.1111/ele.13224 30714287

[bib20] Hedrick BP, Heberling JM, Meineke EK, Turner KG, Grassa CJ, Park DS, Kennedy J, Clarke JA, Cook JA, Blackburn DC et al. 2020 Digitization and the future of natural history collections. BioScience 70: 243–251.

[bib21] Ingram RJ, Levy F, Barrett CL, Donaldson JT 2017 Mining herbaria for clues to the historic prevalence of lily leaf spot disease (*Pseud**o**cercosporella inconspicua*) nn Gray's lily (*Lilium grayi*) and Canada lily (*L. canadense*). Rhodora 119: 163–173.

[bib22] Lacey EA et al. 2017 Climate change, collections and the classroom: Using big data to tackle big problems. Evolution: Education and Outreach 10: 2.

[bib23] Law W, Salick J. 2005 Human-induced dwarfing of Himalayan snow lotus, *Saussurea laniceps* (Asteraceae). Proceedings of the National Academy of Sciences 102: 10218–10220. 10.1073/pnas.0502931102 PMC1177378 16006524

[bib24] LeCun Y, Bengio Y, Hinton G 2015 Deep learning. Nature 521: 436–444. 26017442 10.1038/nature14539

[bib25] Lees DC, Lack HW, Rougerie R, Hernandez-Lopez A, Raus T, Avtzis ND, Augustin S, Lopez-Vaamonde C 2011 Tracking origins of invasive herbivores through herbaria and archival DNA: The case of the horse-chestnut leaf miner. Frontiers in Ecology and the Environment 9: 322–328.

[bib26] Leger EA. 2013 Annual plants change in size over a century of observations. Global Change Biology 19: 2229–2239. 23529770 10.1111/gcb.12208

[bib27] Lendemer J, Thiers B, Monfils AK, Zaspel J, Ellwood ER, Bentley A, LeVan K, Bates J, Jennings D, Contreras D, Lagomarsino L, Mabee P, Ford LS, Guralnick R, Gropp RE, Revelez M, Cobb N, Seltmann K, Aime MC 2019 The extended specimen network: A strategy to enhance US biodiversity collections, promote research and education. BioScience 70: 23–30. 31949317 10.1093/biosci/biz140 PMC6956879

[bib28] Long M, Zhu H, Wang J, Jordan MI 2016 Unsupervised domain adaptation with residual transfer networks. Pages 136–144 in Jordan MI, LeCun Y, Solla SA, eds. Advances in Neural Information Processing Systems.

[bib29] Lorieul T et al. 2019 Toward a large-scale and deep phenological stage annotation of herbarium specimens: Case studies from temperate, tropical, and equatorial floras. Applications in Plant Sciences 7: e01233. 30937225 10.1002/aps3.1233 PMC6426157

[bib30] Love NLR, Park IW, Mazer SJ 2019 A new phenological metric for use in pheno*‐*climatic models: A case study using herbarium specimens of *Streptanthus tortuosus*. Applications in Plant Sciences 7: e11276. 31346508 10.1002/aps3.11276 PMC6636619

[bib31] Meineke EK, Dunn RR, Frank SD 2014 Early pest development and loss of biological control are associated with urban warming. Biology Letters 10: 20140586. 25411378 10.1098/rsbl.2014.0586 PMC4261856

[bib32] Meineke EK, Davis CC, Davies TJ 2018a The unrealized potential of herbaria in global change biology. Ecological Monographs 88: 505–525.

[bib33] Meineke EK, Classen AT, Sanders NJ, Davies JT 2018b Herbarium specimens reveal increasing herbivory over the past century. Journal of Ecology 107: 105–117.

[bib34] Mnih V, Heess N, Graves A, Kavukcuoglu K 2014 Recurrent models of visual attention. Advances in Neural Information Processing Systems. https://papers.nips.cc/paper/5542-recurrent-models-of-visual-attention.pdf.

[bib35] Nelson G, Ellis S. 2018 The history and impact of digitization and digital data mobilization on biodiversity research. Philosophical Transactions of the Royal Society B 374 (art. 20170391). 10.1098/rstb.2017.0391. PMC6282090 30455209

[bib36] Norouzzadeh MS, Nguyen A, Kosmala M, Swanson A, Palmer MS, Packer C, Clune J 2018 Automatically identifying, counting, and describing wild animals in camera-trap images with deep learning. Proceedings of the National Academy of Sciences 115: E5716–E5725. 10.1073/pnas.1719367115 PMC6016780 29871948

[bib37] Park IW, Mazer SJ. 2018 Overlooked climate parameters best predict flowering onset: Assessing phenological models using the elastic net. Global Change Biology 24: 5972–5984. 30218548 10.1111/gcb.14447

[bib38] Parmesan C, Yohe G. 2003 A globally coherent fingerprint of climate change impacts across natural systems. Nature 421: 37–42. 12511946 10.1038/nature01286

[bib39] Primack RB, Miller-Rushing AJ. 2012 Uncovering, collecting, and analyzing records to investigate the ecological impacts of climate change: A template from Thoreau's Concord. BioScience 62: 170–181.

[bib40] Reich PB. 2014 The world-wide “fast-slow” plant economics spectrum: A traits manifesto. Journal of Ecology 102: 275–301.

[bib41] Ronneberger O, Fischer P, Brox T 2015 U-Net: Convolutional networks for biomedical image segmentation. Pages 2204–2212 in Navab N, Hornegger J, Wells W, Frangi A, eds. Medical Image Computing and Computer-Assisted Intervention: MICCAI 2015. Lecture Notes in Computer Science, vol. 9351 Springer.

[bib42] Stucky BJ, Guralnick R, Deck J, Denny EG, Bolmgren K, Walls R 2018 The plant phenology ontology: A new informatics resource for large-scale integration of plant phenology data. Frontiers in Plant Science 9: 517. 29765382 10.3389/fpls.2018.00517 PMC5938398

[bib43] Taylor SD, Meiners JM, Riemer K, Orr MC, White EP 2018 Comparison of large-scale citizen science data and long-term study data for phenology modeling. Ecology 100: e02568. 30499218 10.1002/ecy.2568 PMC7378950

[bib44] Towns J et al. 2014 XSEDE: Accelerating scientific discovery. Computing in Science and Engineering 16: 62–74..

[bib45] Unger J, Merhof D, Renner S 2016 Computer vision applied to herbarium specimens of German trees: Testing the future utility of the millions of herbarium specimen images for automated identification. BMC Evolutionary Biology 16: 248. 27852219 10.1186/s12862-016-0827-5 PMC5112707

[bib46] Willis CW et al. 2017a Old plants, new tricks: Phenological research using herbarium specimens. Trends in Ecology and Evolution 32: 531–546. 28465044 10.1016/j.tree.2017.03.015

[bib47] Willis CW et al. 2017b *CrowdCurio*: An online crowdsourcing platform to facilitate climate change studies using herbarium specimens. New Phytologist 215: 479–488. 28394023 10.1111/nph.14535

[bib48] Wolkovich EM et al. 2012 Warming experiments underpredict plant phenological responses to climate change. Nature 485: 494. 22622576 10.1038/nature11014

[bib49] Wulff F, Schäufele B, Sawade O, Becker D, Henke B, Radusch I 2018 Early fusion of camera and lidar for robust road detection based on U-Net FCN. 2018 IEEE Intelligent Vehicles Symposium (IV) IEEE 1426–1431.

[bib50] Yost JM et al. 2018 Digitization protocol for scoring reproductive phenology from herbarium specimens of seed plants. Applications in Plant Sciences. 6: e1022. 29732253 10.1002/aps3.1022 PMC5851559

[bib51] Zalamea PC, Munoz F, Stevenson PR, Paine CT, Sarmiento C, Sabatier D, Heuret P 2011 Continental-scale patterns of Cecropia reproductive phenology: Evidence from herbarium specimens. Proceedings of the Royal Society B 278: 2437–2445. 21227965 10.1098/rspb.2010.2259 PMC3125618

